# Long lasting pain hypersensitivity following ligation of the tendon of the masseter muscle in rats: A model of myogenic orofacial pain

**DOI:** 10.1186/1744-8069-6-40

**Published:** 2010-07-15

**Authors:** Wei Guo, Hu Wang, Shiping Zou, Feng Wei, Ronald Dubner, Ke Ren

**Affiliations:** 1 Department of Neural and Pain Sciences, Dental School; & Program in Neuroscience, University of Maryland, Baltimore, MD 21201, USA

## Abstract

**Background:**

A major subgroup of patients with temporomandibular joint (TMJ) disorders have masticatory muscle hypersensitivity. To study myofacial temporomandibular pain, a number of preclinical models have been developed to induce myogenic pain of the masseter muscle, one of the four muscles involved in mastication. The currently used models, however, generate pain that decreases over time and only lasts from hours to weeks and hence are not suitable for studying chronicity of the myogenic pain in TMJ disorders. Here we report a model of constant myogenic orofacial pain that lasts for months.

**Results:**

The model involves unilateral ligation of the tendon of the anterior superficial part of the rat masseter muscle (TASM). The ligation of the TASM was achieved with two chromic gut (4.0) ligatures via an intraoral approach. Nocifensive behavior of the rat was assessed by probing the skin site above the TASM with a series of von Frey filaments. The response frequencies were determined and an EF_50 _value, defined as the von Frey filament force that produces a 50% response frequency, was derived and used as a measure of mechanical sensitivity. Following TASM ligation, the EF_50 _of the injured side was significantly reduced and maintained throughout the 8-week observation period, suggesting the presence of mechanical hyperalgesia/allodynia. In sham-operated rats, the EF_50 _of the injured side was transiently reduced for about a week, likely due to injury produced by the surgery. Somatotopically relevant Fos protein expression was indentified in the subnucleus caudalis of the spinal trigeminal sensory complex. In the same region, persistent upregulation of NMDA receptor NR1 phosphorylation and protein expression and increased expression of glial markers glial fibrillary acidic protein (astroglia) and CD11b (microglia) were found. Morphine (0.4-8 mg/kg, s.c.) and duloxetine (0.4-20 mg/kg, i.p.), a selective serotonin-norepinephrine reuptake inhibitor, produced dose-dependent attenuation of hyperalgesia.

**Conclusions:**

Ligation injury of the TASM in rats led to long-lasting and constant mechanical hypersensitivity of myogenic origin. The model will be particularly useful in studying the chronicity of myogenic pain TMJ disorders. The model can also be adapted to other regions of the body for studying pathology of painful tendinopathy seen in sports injury, muscle overuse, and rheumatoid arthritis.

## Background

Temporomandibular joint (TMJ) disorders are often associated with chronic pain conditions and therefore a severe health problem. A major subgroup of patients with TMJ disorders have masticatory muscle hypersensitivity [1,2]. To study myofacial temporomandibular pain, a number of models have been developed in the recent decades to induce myogenic pain of the masseter muscle, one of the four muscles involved in mastication. The algesic agents hypertonic saline, glutamate, serotonin, capsaicin and nerve growth factor are injected into the masseter muscle in humans [3-8]. The inflammatory agent complete Freund's adjuvant (CFA) is injected into the rat masseter to produce inflammatory hyperalgesia [9-11]. Injection of glutamate, capsaicin or mustard oil into the masseter muscle produces peripheral sensitization and mechanical hyperalgesia in rats [12-15]. These models have generated important information on neural mechanisms of persistent pain associated with TMJ disorders.

The currently used models, however, are not suitable for studying chronicity of the myogenic pain in TMJ disorders, which is a deteriorating and devastating problem for the patients. Most algesic agents when injected into the masseter muscle only induce an effect that lasts for minutes to hours [3-6,12,13]. Relative long lasting hyperalgesia can be induced by injecting nerve growth factor into the masseter in humans [7] and CFA into the rat masseter [9,10], but the hyperalgesia decreases over time. Nevertheless, the hyperalgesia following the nerve growth factor and CFA treatment is resolved within 1-3 weeks, which is unlike the long persistency of chronic pain seen in TMJ disorders.

Here we report a model of myogenic orofacial pain that lasts for months. The model involves unilateral ligation of the tendon of the anterior superficial part of the rat masseter muscle (TASM), which is a distinct structure arising from the lateral surface of the maxilla just posterior to the suture of the maxilla and premaxilla [16]. Constriction injury was produced by the ligation, leading to long-lasting and stable mechanical allodynia/hyperalgesia that can be assessed through the cutaneous site overlying the tendon.

## Methods

### Animals

Male Sprague-Dawley rats, ≈ 8-week old at the time of surgery, were used (225-250 g, Harlan, Indianapolis, IN). Ligation of the TASM was achieved via an intraoral approach. Animals were anesthetized with pentobarbital sodium (50 mg/kg i.p.). The rat was fixed on a table and the head was supine. The mouth of the rat was kept open with a retractor. On the left side, a three-mm long incision was made posterior-anteriorly along but just lateral to the gingivobuccal margin in the buccal mucosa, beginning immediately next to the first molar. The TASM was gently freed from surrounding connective tissues and clearly visualized. The tendon was tied with two chromic gut (4.0) ligatures, 2-mm apart. The wound was checked for hemostasis and the incision closed with three 4.0 silk sutures. The sham-operated rats received the same procedure except that the tendon was not ligated. Changes in gross behavior and body weight in operated rats were monitored and compared with that in naive rats. Standard hematoxylin and eosin (EMS, Hatfield, PA) staining was performed to examine histological changes of the TASM after ligation, including infiltration of immune cells and morphology of the tendon tissue. For time course of hyperalgesia, 14 rats were used for the TASM ligation and sham groups, respectively. For local anesthesia and analgesic drugs experiments, 5 rats were used for each experimental group. For immunohistochemistry and western blot experiments, 3-4 rats were used for each group. All experiments were carried out in accordance with the National Institute of Health Guide for the Care and Use of Laboratory Animals (NIH Publications No. 80-23) and approved by the University of Maryland Dental School Institutional Animal Care and Use Committee. All efforts were made to minimize the number of animals used and their suffering.

### Behavioral testing

All behavioral tests were conducted under blind conditions as described elsewhere [9,17]. A series of calibrated von Frey filaments were applied to the skin above the TASM. An active withdrawal of the head from the probing filament was defined as a response. Each von Frey filament was applied 5 times at intervals of a few sec. The response frequencies [(number of responses/number of stimuli) × 100%] to a range of von Frey filament forces were determined and a stimulus-response frequency (S-R) curve plotted. After a non-linear regression analysis, an EF_50 _value, defined as the von Frey filament force (g) that produces a 50% response frequency, was derived from the S-R curve (Prism, GraphPad). We used EF_50 _values as a measure of mechanical sensitivity. A leftward shift of the S-R curve [9,18,19], resulting in a reduction of EF_50_, occurred after ligation of the TASM. This shift of the curve suggests the presence of mechanical hyperalgesia and allodynia since there was an increase in response to suprathreshold stimuli and a decreased response threshold for nocifensive behavior.

### Immunohistochemistry

At 3 d, 2 w and 8 w after ligation, rats were behaviorally tested to identify tendon ligation-induced mechanical hyperalgesia/allodynia, and then deeply anesthetized with pentobarbital sodium (100 mg/kg, i.p.) and perfused transcardially with 4% paraformaldehyde in 0.1 M phosphate buffer at pH 7.4. Naive rats and rats at 3 d and 2 w after sham operation were also perfused. A tissue block including a 2-mm segment at the caudal trigeminal subnucleus caudalis (Vc, 3-5 mm caudal to the obex) was dissected. This tissue block includes the caudal laminated Vc and C1 upper cervical spinal cord, the major component of the medullary dorsal horn. The brain stem block was post-fixed and transferred to 25% sucrose (w/v) for cryoprotection. Thirty micrometer-thick coronal sections of the brainstem were cut with a cryostat at -20°C. Free-floating sections were incubated with antibodies overnight with 1-3% relevant normal sera. For Fos-like immunoreactivity (LI), after washes in PBS, the sections were incubated with biotinylated secondary IgG (Vector, 1:400) and then with avidin and biotinylated horseradish peroxidase complex (Vector, 1:100). The tissue sections are finally reacted with 0.05% diaminobenzidine dihydrochloride (Sigma) in 0.1 M PB containing 0.003% hydrogen peroxide for 3-6 min. For fluorescence staining (phosphorylation at serine 896 of the NR1 subunit, P-NR1; GFAP, glial fibrillary acidic protein; CD11b, cluster of differentiation 11b; NeuN), the sections were incubated with Streptavidin-Alexa Fluor 568 or 488 (1:600, Molecular Probes, Eugene, OR) for 1 h. The sections were then incubated for 2 h in solutions containing species-specific secondary antibodies coupled to Cy3, Alexa 568 or Alexa 488. After washes in PBS, all sections were mounted on gelatin-coated slides and coverslipped with Vectashield (Vector Laboratories). Images were collected sequentially using a Nikon fluorescence microscope and a charge-coupled device camera controlled by SPOT software. Adobe Photoshop (version CS4) was used for image cropping and adjustment. Immunostaining control studies were performed by omission of the primary or secondary antibodies, and by adsorption with an excess (10 μg/ml) of the respective antigens.

### Western blot

The rats were anesthetized with isoflurane (3%) and quickly decapitated. The same block of caudal brainstem tissues as that for immunohistochemistry was collected. The tissue block was turned coronally and the superficial portion of the Vc was harvested by taking punches with a 15-gauge puncture needle. The tissues were homogenized in solubilization buffer (50 mM Tris.HCl, pH8.0; 150 mM NaCl, 1 mM EDTA, 1% NP40, 0.5% deoxycholic acid, 0.1% SDS, 1 mM Na3VO4, 1 U/ml aprotinin, 20 μg/ml leupetin, 20 μg/ml pepstatin A). The homogenate was centrifuged at 20,200 × g for 10 min at 4°C. The supernatant was removed. The protein concentration was determined using a detergent-compatible protein assay with a bovine serum albumin standard. Each sample contains proteins from one animal. The proteins (50 μg) were separated on a 7.5% SDS-PAGE gel and blotted to a nitrocellulose membrane (Amersham Biosciences, Arlington Heights, IL). The blots were blocked with 5% milk in tris-buffered saline (TBS) buffer and then incubated with the respective antibody. The membrane was washed with TBS and incubated with anti-goat or mouse IgG (1:3000, Santa Cruz Biotechnology, Santa Cruz, CA). The immunoreactivity was detected using Enhanced Chemiluminescence (ECL, Amersham). The loading and blotting of the amount of protein was verified by reprobing the membrane with anti-β-actin antiserum (Sigma) and with Coomassie blue staining.

### Drugs and antibodies

The following drugs and antibodies were purchased from commercial sources: Morphine sulfate salt pentahydrate (Sigma, St. Louis, MO), dissolved in physiological saline (0.09%); lidocaine (Henry Schein, Melville, NY); anti-Fos antibody (EMD Biosciences, San Diego, CA) anti-GFAP, anti-NR1, anti-P-ser896 NR1 and anti-NeuN antibodies (Millipore-Chemicon, Temecula, CA), and anti-CD11b (clone OX-42) (AbD Serotec, Raleigh, NC). Duloxetine (dissolved in 50% H_2_O/50% hydroxypropyl-β-cyclodextrin, Sigma) was provided by Eli Lilly and Company (Indianapolis, IN). Drug doses are expressed as milligram salt per kilogram body weight (mg/kg). The injection volume was 1 ml/kg for systemic administration.

### Data analysis

Data are presented as mean (95% confidence interval) for EF_50_s and mean ± S.E.M. for other data. Statistical comparisons were made by the use of ANOVA with Fisher's PLSD test for post-hoc analysis. Non-parametric statistics were used for comparing EF_50_s (Kruskal-Wallis, Mann-Whitney). Alternate sections were used for quantifying Fos-positive neurons in the Vc-C1. Means of three sections from each animal were used to derive group means. For western blot analysis, the ECL-exposed films or gel images were digitized and densitometric quantification of immunoreactive cDNA bands was carried out using UN-SCAN-IT gel (ver. 5.3, Silk Scientific Inc., Orem, UT). The relative protein or mRNA levels were obtained by comparing the respective specific band to the β-actin control from the same membrane or gels. The deduced ratios were further normalized to that of the naive rats on the same membrane and illustrated as percentage of the naïve controls. For animals that were subject to repeated testing, ANOVA with repeated measures was used with time as a within animal effect. P < 0.05 was considered significant for all cases.

## Results

### Ligation injury of the tendon of the masseter muscle

**Figure 1:**
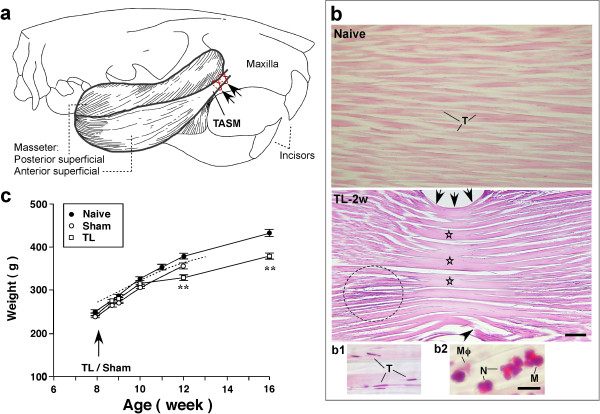
**Ligation of the masseter tendon of the rat**. **a**. Drawing of rat skull and masseter muscle. The tendon (TASM) attachment of the anterior superficial portion of the masseter muscle to the right maxillary bone and the site of ligation with two ligatures are shown (arrows). **b**. Hematoxylin and eosin staining illustrating histology of the masseter tendon in naive (top) and TASM-ligated rats (TL-2w). Naive rat exhibited normal organized collagen fibers and uniformly distributed tenocytes (T, also see b1). Arrows in TL-2w panel indicate site of ligation injury. Note the depressed region produced by ligature (arrows). Compared to the naive rat, adhesive bands of collagen fibers were formed at the level of ligation (stars). Disruption of major collagen fiber bundle was found (arrowhead). Clusters of purple stained inflammatory cells mixed with tenocytes were seen (circle). Scale = 0.2 mm. **b1, b2**. High power photomicrograph images showing examples of tenocytes in the normal tendon from a naive rat (b1) and examples of inflammatory cells are shown (b2). Mϕ, macrophage; M, monocyte; N, neutrophil. Scale = 0.02 mm. **c**. Comparison of body weight of the rats. Dashed line is adapted from Growth Chart of Harlan Sprague Dawley rat [20]. Note that the TL group had slowed growth at 12-16-week (4-8 weeks after injury) (p < 0.01, vs. naive rats). Error bars represent S.E.M.

The rat masseter muscle consists of four parts: anterior and posterior superficial, and anterior and posterior deep [16]. The anterior superficial part of the rat masseter muscle has a strong slender tendon that is easily approachable from the intraoral space (Fig. 1). We took advantage of this distinct anatomical feature to produce ligation injury of the TASM. Two chromic gut ligatures were placed and tied around to the TASM as indicated in Fig. 1a. Hematoxylin and eosin staining of the TASM shows points of depression by the ligatures and disrupted and disarrayed collagen fiber bundles (Fig. 1b). Compared to naive rats, adhesive bands of collagen fibers formed at the level of ligation (Fig. 1b). There was infiltration of inflammatory cells into the injured tendon, examined at 3 d and 2 w after ligation. Clusters of inflammatory cells mixed with tenocytes were found adjacent to the traumatized segment (Fig. 1b). Infiltrated leukocytes were also seen in the muscle adjacent to the tendon (not shown). At 8 w after ligation, no inflammatory cells were observed in the ligated tendon and there is scar formation in the injured area with rounded tenocytes clusters (not shown).

To assess the effect of intraoral surgery on general health of the animals, the growth rate of the animals was compared between different groups by monitoring body weight. We first determined that the growth curve of incoming naive rats (n = 6) was consistent with the growth chart of Sprague Dawley rats [20] (Fig. 1c). The growth curves were then compared between naive, tendon-ligated (n = 14) and sham-operated (n = 14) animals. The results indicate that the operated rats had similar growth rate with that of the naive rats within two weeks after the surgery. At 4-8 weeks after surgery, rats receiving tendon ligation showed slowed body growth (p < 0.01) while the sham-operated rats were able to maintain normal body weight as compared to naive rats and the general Sprague-Dawley population (Fig. 1c). These results suggest that, with delayed onset, the tendon ligation injury had a long-lasting impact on the rat's growth. The rats groomed the area above the tendon ligation less within a few days of surgery but otherwise displayed normal locomotor activity, explored their environment, and interacted with other rats.

### Persistent behavioral hyperalgesia after tendon ligation

**Figure 2:**
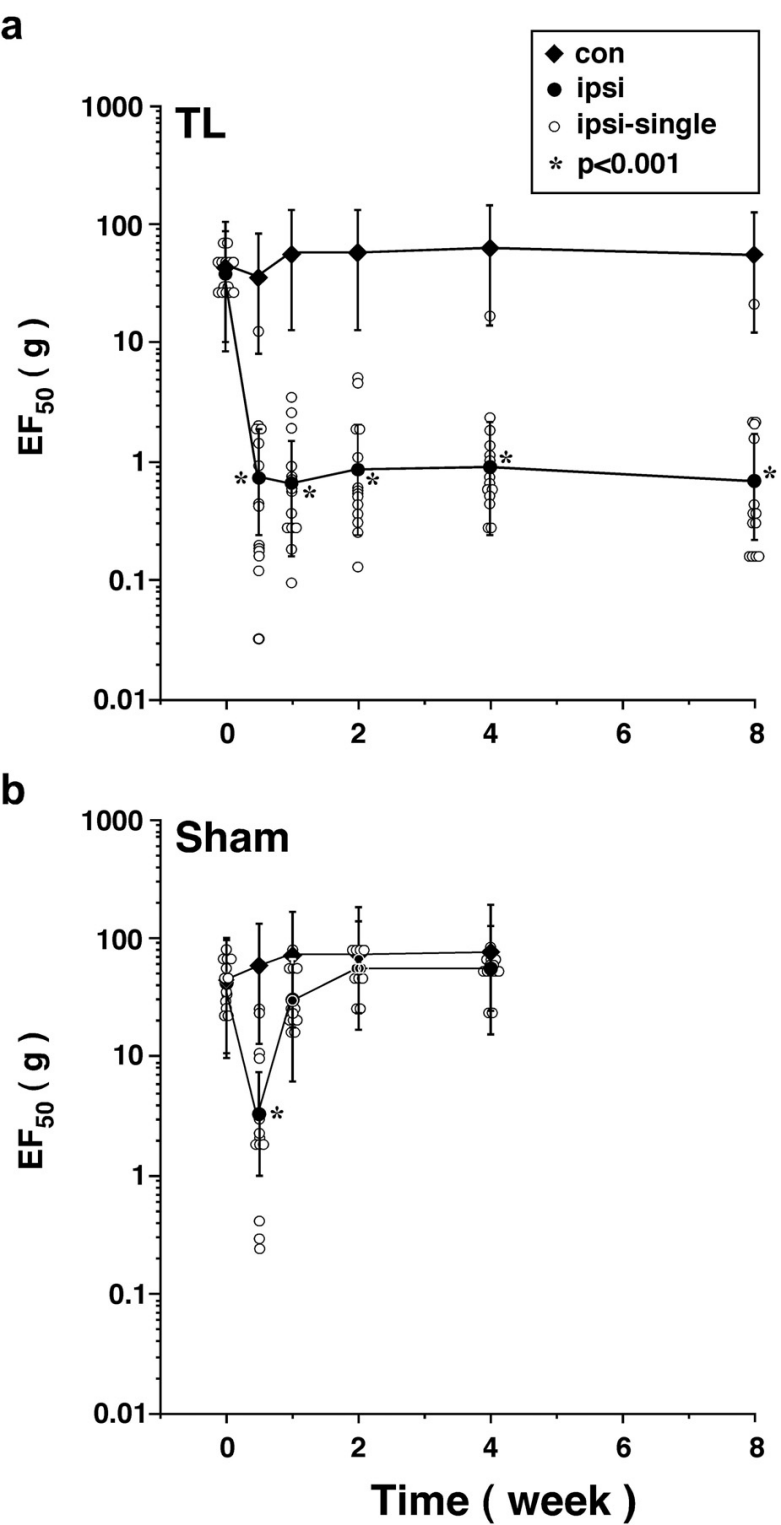
**Mechanical hyperalgesia/allodynia following ligation of the masseter tendon**. The orofacial skin site above the TASM was probed with a series of von Frey microfilaments. The EF_50_s were derived from the respective stimulus-response frequency function curves and are plotted against time after surgery. A significant reduction of EF_50 _indicates increased mechanical sensitivity, or mechanical hyperalgesia and allodynia. Note log scales for the ordinate. Filled circles show group means and open circles indicate individual EF_50 _of the side ipsilateral to injury. Error bars are 95% confidence intervals of EF_50_s. **a**. Rats receiving ligation of the TASM (n = 14). A significant reduction of EF_50 _indicating significant mechanical allodynia occurred ipsilateral to injury at 3 d and was maintained through the 8-week period. There was no change in EF_50 _on the equivalent contralateral site. **b**. Sham-operated rats showed a temporary reduction of EF_50 _at 3 d ipsilateral to intraoral procedure (n = 14). *, p < 0.01 vs. contralateral site.

Following TASM ligation, the EF_50 _of the injured side was significantly reduced (Fig. 2a), indicating the development of mechanical hyperalgesia/allodynia. The reduction of EF_50 _was significant (p < 0.001, n = 14) at the first test (3 d post ligation) and the hyperalgesia/allodynia maintained throughout the observation period up to 8 weeks. There were no significant changes in EF_50 _on the contralateral side, suggesting that the effect of ligation was limited to the site of injury. In sham-operated rats (n = 14), the EF_50 _on the injured side was reduced initially after surgery. The reduction of EF_50 _in the sham rats was smaller compared to the tendon-ligated rats and returned to the baseline level by 7-10 days. The small and short-lasting hypersensitivity after sham operation was likely due to tissue injury produced by the surgical procedure, similar to that seen in the sham operation of infraorbital nerve injury [21].

**Figure 3:**
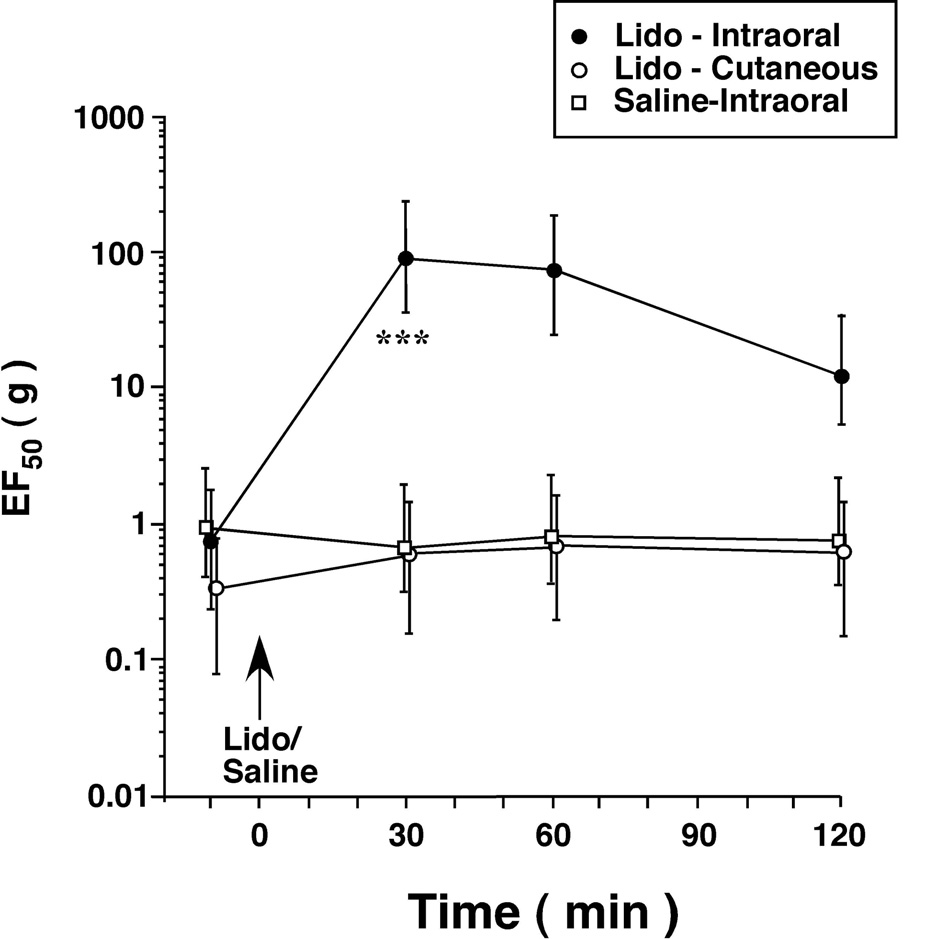
**Effect of local anesthesia on mechanical allodynia and hyperalgesia after TASM ligation**. To verify allodynia/hyperalgesia related to tendon injury, local anesthesia was induced in the intraoral site around the TASM or the overlying skin ipsilaterally by lidocaine (4%, 0.05-0.2 ml) in rats receiving tendon ligation and having developed allodynia/hyperalgesia. Compared to saline-treated rats (Saline-Intraoral, n = 5), allodynia/hyperalgesia was significantly attenuated when lidocaine was infiltrated into the TASM site (Lido-Intraoral, n = 5) at 30 min after lidocaine (***, p < 0.001, vs. saline). Local anesthesia of the skin overlying TASM (Lido-Cutaneous, n = 5) did not affect hyperalgesia. Note that some symbols are slightly shifted horizontally for clarity. Error bars represent 95% confidence intervals.

We assessed the mechanical sensitivity of the tendon through probing the overlying skin. Although deep tissue primary afferents are activated by this mechanical pressure, a problem with this approach is that cutaneous afferents are also stimulated and may contribute to the pain-pressure threshold [22]. To verify deep origin of hyperalgesia after the tendon injury, we induced anesthesia by infiltrating the local anesthetic lidocaine (4%, 0.05-0.2 ml) into tissues surrounding the TASM or the overlying skin in rats that had developed hyperalgesia after tendon ligation. The hyperalgesia was largely attenuated, as shown by a significantly increase in EF_50 _(p < 0.001, n = 5), when the TASM was anesthetized as compared to saline-treated rats (Fig. 3). The effect of local anesthesia started to wear off after 60 min (Fig. 3). In contrast, the tendon injury-induced hyperalgesia was not affected when the overlying skin testing site was anesthetized (n = 5) (Fig. 3). Thus, a reduction of EF_50 _at the testing site in tendon-ligated rats reflects tenderness and increased sensitivity of deep tissue. This is consistent with previous results that masseter hyperalgesia was detectable when the overlying skin site was anesthetized [23].

### Neuronal activation in the spinal trigeminal nucleus

**Figure 4:**
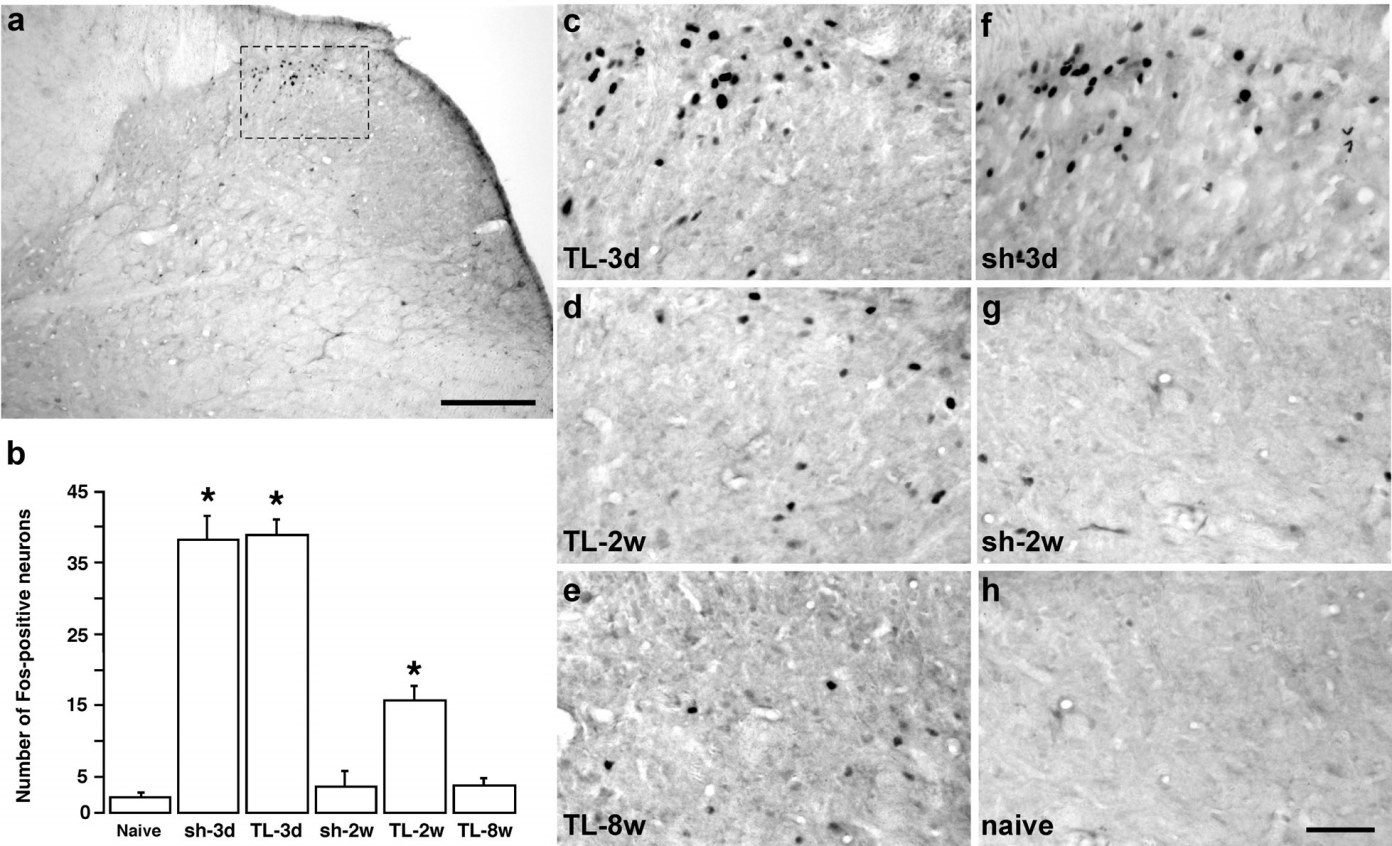
**Fos protein expression in the spinal trigeminal nucleus after TASM ligation**. **a**. Low power photomicrograph of a section of the upper cervical dorsal horn illustrating induction of Fos-LI in superficial laminae (dashed rectangle). Scale = 0.2 mm. **b**. Histogram illustrating mean number of Fos-positive neurons in superficial laminae of Vc-C1 ipsilateral to TASM ligation (TL). Naive rats were used as a control. *, p < 0.05 vs. naive, n = 3/group. Error bars represent S.E.M. **c-h**. Examples of Fos-LI. All fields were taken and enlarged from a portion of medial-intermediate superficial dorsal horn as shown by the rectangle in **a**. Scale = 0.05 mm.

We used Fos-LI to assess neuronal activation in the trigeminal spinal nucleus after ligation of the TASM. Fos-immunoreactive reaction product appeared as distinctive light to dark brown staining in cellular nuclei. In the laminated subnucleus caudalis and upper cervical dorsal horn, Fos-positive cells were mainly clustered in the medial-intermediate portion of superficial laminae on the side ipsilateral to ligation (Fig. 4a). There were no significant increases in Fos-LI in the Vc-C1 contralateral to the injury (not shown). The distribution of Fos-LI is consistent with somatotopy of the V3 input with a small V2 component. Few Fos-labeled neurons were found in laminae III/IV and V/VI. The number of Fos-positive cells in the superficial laminae was quantified. Compared to naive animals (n = 3) where very few Fos-positive cells (< 5 cells/section) were seen (Fig. 4b,h), ligation of TASM and sham injury induced a significant increase in the number of Fos-labeled cells at 3 d after operation (p < 0.05, n = 3) (Fig. 4b,c,f). At 2 weeks after ligation, there was still a significant increase in Fos-LI, although it was reduced in magnitude compared to the 3 d time point (n = 3). The Fos-LI returned to the naive level at 2 weeks in sham-operated animals (Fig. 4b,g) (n = 3). At 8 weeks after tendon ligation (n = 3), Fos-LI was at the naive level in ligated animals (Fig 4b,e).

### Activation of NMDA receptors and glia

**Figure 5:**
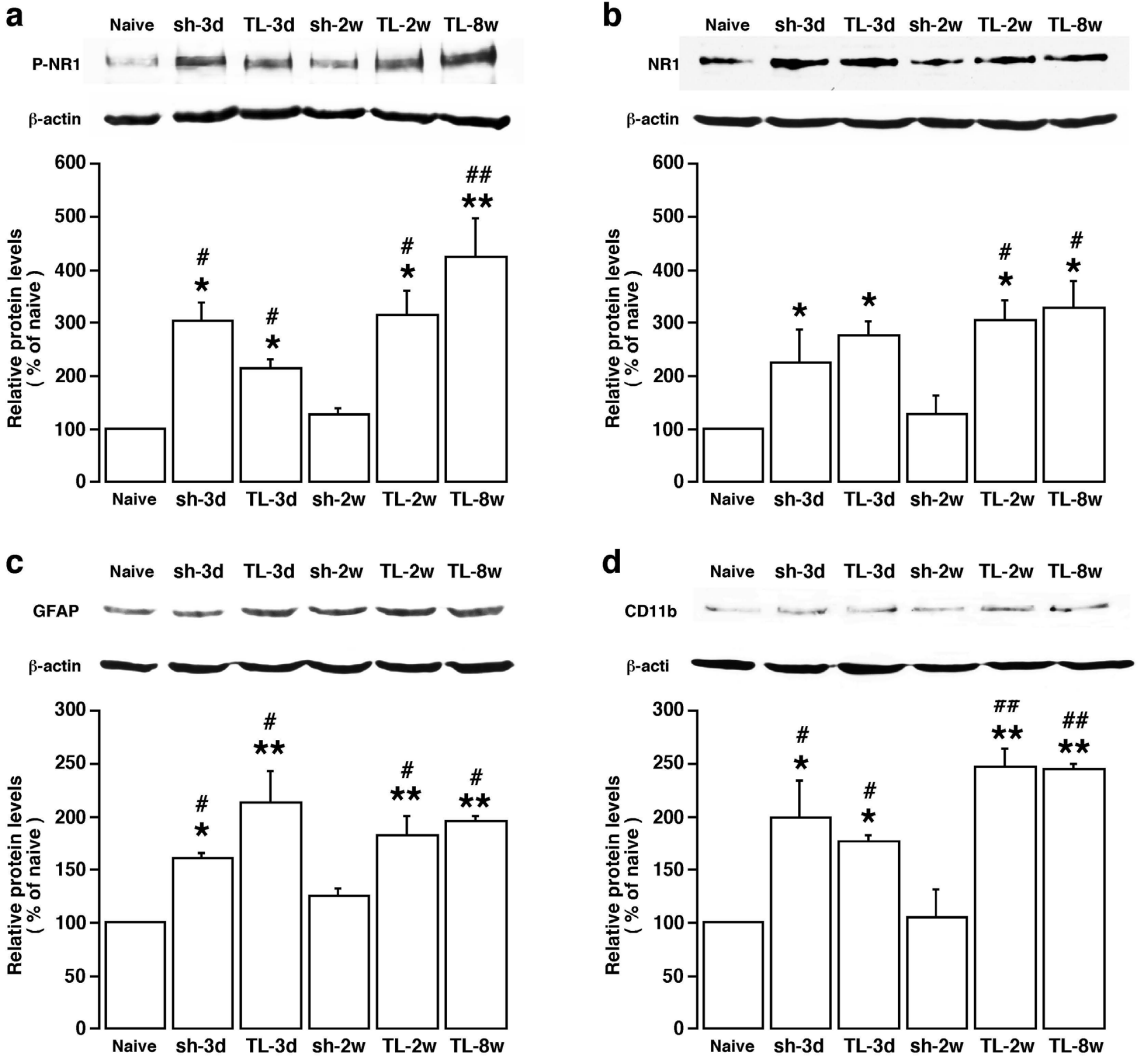
**Western immunoblot illustrating TASM-ligation-induced upregulation of NMDA receptors and glial markers**. The dorsal Vc-C1 was punched out at various time points and total proteins isolated. Beta-actin was used as a loading control. An example of the blot is shown on top and the relative protein levels are shown in the bottom histogram. Note the increased levels of P-NR1, NR1 protein, GFAP and CD11b in TL rats at 3 d, 2w and 8w. Sham rats only showed the increases at 3 d after the procedure. *, p < 0.05; **, p < 0.01, vs. naive rats. #, p < 0.05; ##, p < 0.01, vs. sh-2w. N = 3-4 for each time point. Error bars represent S.E.M.

**Figure 6:**
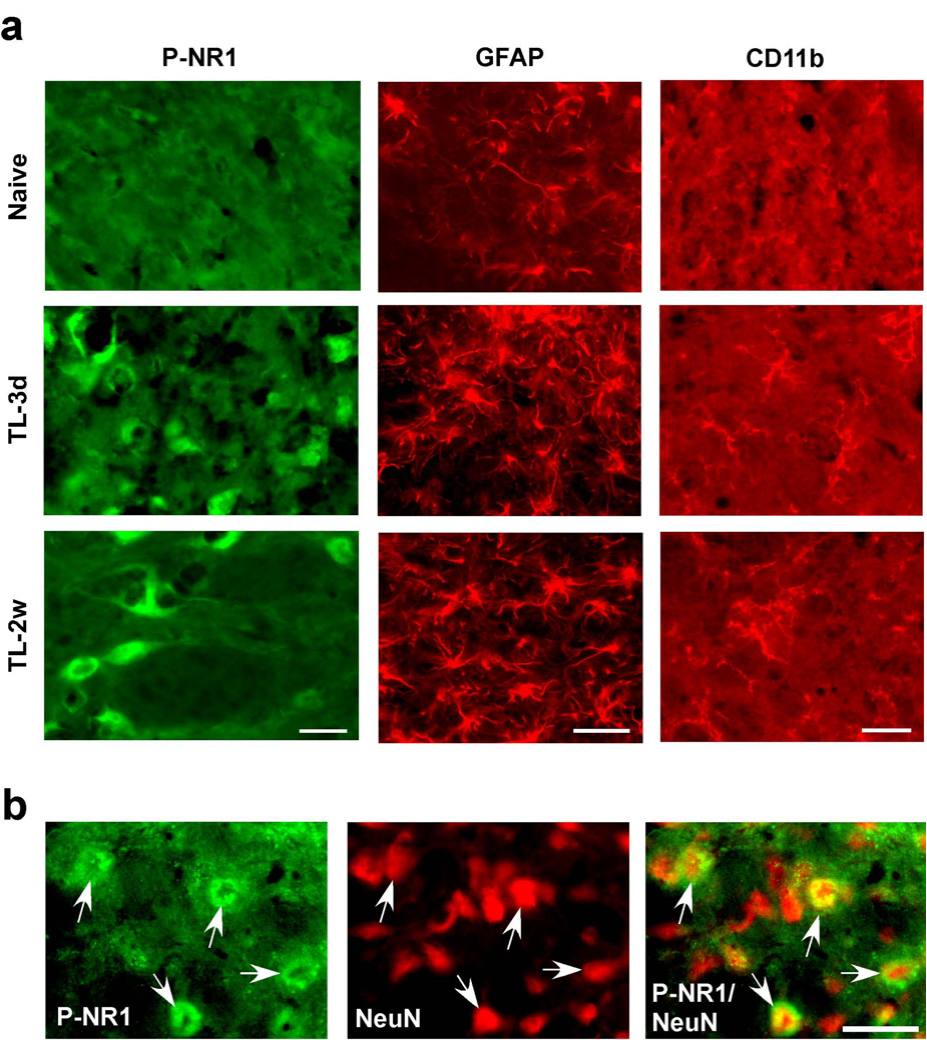
**Immunofluorescence staining of phosphoserine896-NR1 (P-NR1), GFAP and CD11b in naive and TASM-ligated (TL) rats**. **a**. Note increased immunostaining at 3 d and 2w after tendon ligation, suggesting NMDA receptor activation and glial hyperactivity. Scale = 0.025 mm. **b**. Localization of P-NR1 in Vc neurons at 3 d after tendon ligation. P-NR1 is shown as green fluorescence (Alexa Fluor 488, left). NeuN is visualized with Cy3 (red, middle). Overlap of left and middle panels reveals cells that exhibit double fluorescence of NeuN (nucleus) and P-NR1 (cytoplasm) in the same neurons (right). Examples of double-labeled neurons are indicated by arrows. Scale = 0.05 mm

NMDA receptor phosphorylation has been widely accepted as an indication of synaptic plasticity and correlates with the time course of persistent pain [24]. Tissue injury induces NMDA receptor activation involving phosphorylation of the NR1 and NR2 subunits in the spinal dorsal horn [18,25,26]. The changes in phosphorylated NR1, the obligatory subunit of the NMDA receptor, were quantified by Western blot (n = 3/4/group) in tissues taken from the same region that showed increased Fos-LI after tendon ligation. The results indicate that the levels of P-NR1 increased by 200-400% of the naive level in rats receiving tendon injury (p < 0.05-0.01) (Fig. 5a). The increase in P-NR1 in sham rats was significant at 3 d (p < 0.05, n = 3) but there was no significant change in P-NR1 at 2 w after sham surgery (Fig. 5a). There was also a time-dependent upregulation of the NR1 protein levels (Fig. 5b). The neuronal localization of P-NR1 in Vc-C1 was verified with immunofluorescence. P-NR1-like staining was mainly in the cytoplasm of neurons (Fig. 6, also see Guo 07). Compared to the naive rat, there was an increase in P-NR1 immunostaining in the Vc-C1 superficial dorsal horn after tendon injury (Fig. 6a, left column: TL-3 d and 2w). Sham-operated rat also showed an increased P-NR1 immunostaining at 3 d (not shown). Double immunostaining of P-NR1 with NeuN confirmed that the increased NMDA receptor phosphorylation was in neurons (Fig. 6b).

New evidence indicates that non-neuronal components of the nervous system facilitate synaptic plasticity and participate in chronic pain mechanisms [27]. Astroglia and microglia, the two major glial cell types in the CNS, exhibit an hyper-reactive phenotype after peripheral injury and are believed to play a role in pain hypersensitivity. We examined whether masseter tendon injury induced glial hyperactivity in the spinal trigeminal nucleus. The increase in GFAP, a marker of astrocyte, was quantified by western blot. The tissues from the Vc-C1 dorsal horn ipsilateral to surgery were punched out and total proteins isolated. As shown in Fig. 5c, the increase in GFAP levels was seen at 3 d, 2 w and 8 w after ligation (p < 0.01). Sham-operated rats showed a small increase in GFAP at 3 d (p < 0.05, n = 3) but not at 2 w after surgery (Fig. 5c). The masseter tendon ligation also upregulated the levels of CD11b, a marker of activated microglia. Western blot analysis showed that the pattern of increase in CD11b was similar to that of GFAP (Fig. 5d). Utilizing GFAP immunostaining, reactive astroglia were clearly seen in the Vc-C1 after tendon ligation as compared to naive rats (Fig. 6a, GFAP: TL-3 d and 2w). There was also an increase in CD11b immunostaining after tendon ligation (Fig. 6a, CD11b: TL-3 d and 2w).

### Attenuation of hyperalgesia by analgesics

**Figure 7:**
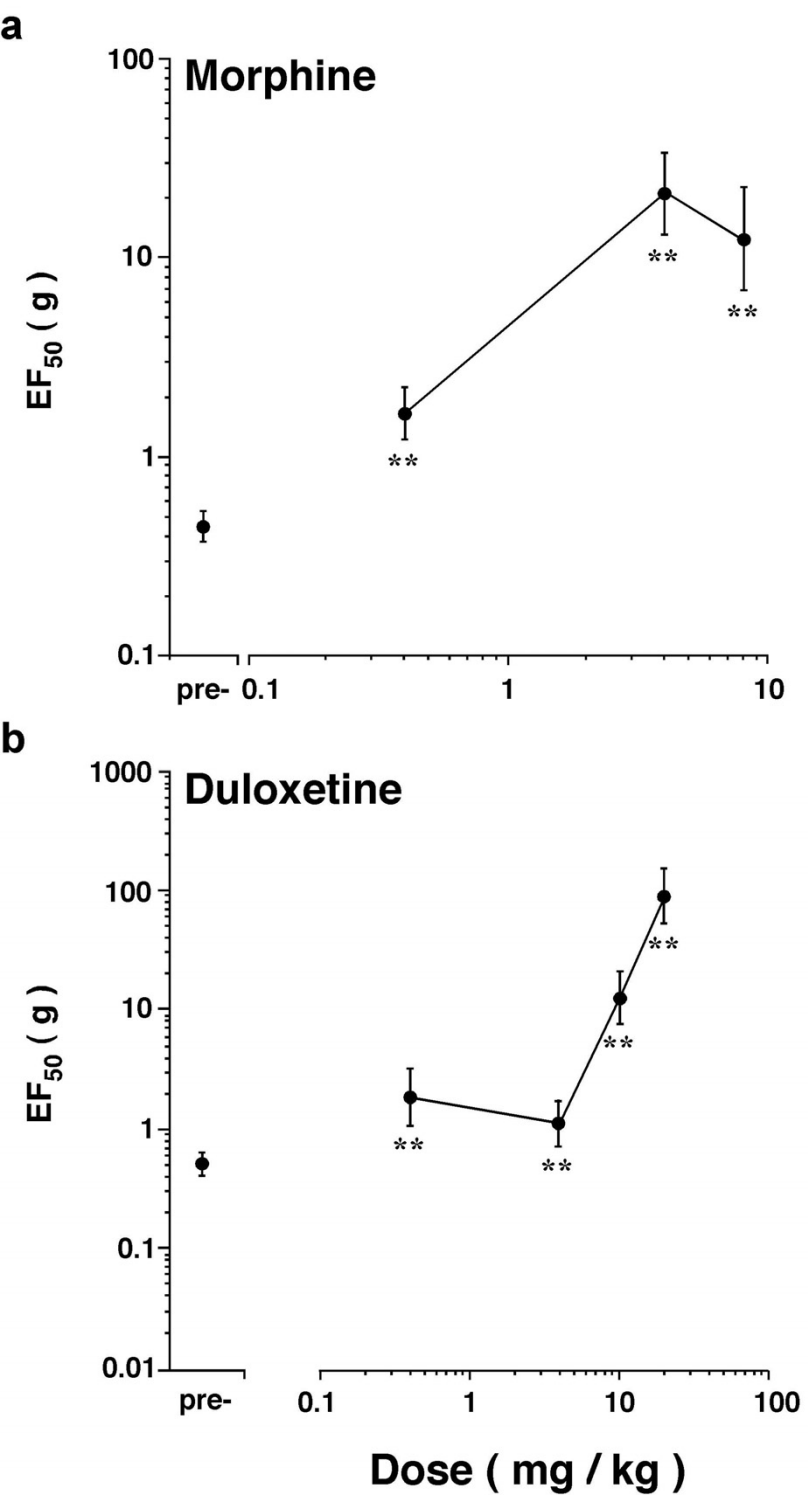
**Effects of morphine and duloxetine on EF_50_s of rats at 8 weeks after ligation of the TASM**. The EF_50_s were below 1 g prior to administration of drugs, indicating the presence of mechanical allodynia. **a**. Morphine was injected s.c. at 0.4, 4 and 8 mg/kg (1 ml/kg, n = 5 per dose). Morphine produced significant and dose-dependent increases in EF_50_s tested at 45 min after injection (ANOVA, P < 0.01). **b**. Duloxetine was injected i.p. at 0.4, 4, 10 and 20 mg/kg (1 ml/kg, n = 5 per dose). Administration of duloxetine produced significant and dose-dependent increases in EF_50_s tested at 60 min after injection (ANOVA, P < 0.01).

We finally verified the sensitivity of the TASM injury model to morphine and duloxetine, the two agents that are clinically used as analgesics and commonly used in preclinical studies [28,29]. The effects of morphine and duloxetine were evaluated in rats at 8 weeks after ligation of the TASM when persistent mechanical hyperalgesia/allodynia had developed (Fig. 2a). Morphine was injected s.c. at 0.4, 4 and 8 mg/kg (1 ml/kg, n = 5 per dose). As shown in Fig. 7a, subcutaneous administration of morphine produced significant and dose-dependent increases in EF_50_s tested at 45 min after injection (ANOVA, P < 0.01). Compared to pre-morphine EF_50 _of 0.45 g (95% confidence interval: 0.38-0.53 g), 4 mg/kg of morphine elevated EF_50 _to 20.9 g (13.1-33.4 g) (p < 0.01). The EF_50 _returned to the pre-morphine level at 90 min after injection. These results indicate that morphine produced a moderate antihyperalgesic effect in the tendon injury model. There was also a significant increase in EF_50_s on the equivalent contralateral site after injection of 4 mg/kg (p < 0.05) and 8 mg/kg (p < 0.01) morphine, confirming the analgesic effect of morphine.

We next studied the effect of duloxetine, a serotonin-norepinephrine reuptake inhibitor. Duloxetine was injected i.p. at 0.4, 4, 10 and 20 mg/kg (1 ml/kg, n = 5 per dose). As shown in Fig. 7b, intraperitoneal administration of duloxetine produced significant and dose-dependent increases in EF_50_s tested at 60 min after injection (ANOVA, P < 0.01). Compared to pre-duloxetine EF_50 _of 0.54 g (0.44-0.66 g), 20 mg/kg of duloxetine elevated EF_50 _to 95.1 g (56.6-160.0 g) (p < 0.01). There was no significant increase in EF_50_s on the equivalent contralateral site after duloxetine.

## Discussion

The major finding of the present study is that simple ligation of one tendon of the rat masseter muscle, TASM, led to long lasting and constant mechanical hyperalgesia/allodynia. The hyperalgesia/allodynia was robust, occurred as early as 3 d after ligation and was maintained throughout the 8-week observation period. Correlated and somatotopically relevant neurochemical changes were identified in the spinal trigeminal nucleus after ligation of the TASM. There was neuronal activation, as indicated by induced Fos protein-LI in superficial laminae of Vc-C1 where trigeminal nociceptive input is processed and relayed. NMDA receptor expression and phosphorylation were upregulated, as well as GFAP and CD11b, markers of astroglia and microglia, respectively. Clinically used analgesics morphine and duloxetine attenuated hyperalgesia/allodynia after TASM ligation, confirming the feasibility of this model in evaluating new treatment approaches for pain management in humans.

Chronic myogenic TMJ pain in humans often involves muscles of mastication, among which masseter muscle dysfunction involving its tendinous origin is frequently seen [30,31]. Clinically, inflammation of the masseter muscle at its attachment to the zygomatic arch is called tenomyositis or tendomyositis, so named as human masseter muscle fibers and layers of tendon alternate at their attachment to the bone [30]. Similarly, chronic musculo-tendinous pain of other body regions is often related to inflammation of the tendon, or tendinitis (tendonitis). It is also noted that chronic pain involving tendon conditions including injury is not necessarily accompanied by inflammatory responses [32,33]. For example, in a histopathological study of the cases of tendon rapture in humans, 97% of samples did not show signs of inflammation [34]. Currently, the term 'tendinopathy' is used to describe conditions of tendon pathology, often with chronic pain, without alluding to underlying pathology [33,35]. Our histopathology results show that inflammatory cells infiltrated into the tendon at 2 weeks after ligation, indicating the presence of inflammation. There was also signs of inflammation in the muscle adjacent to the tendon, suggesting that the observed hyperalgesia/allodynia also involve a muscle component. The inflammation of the tendon/muscle may explain the initiation of hyperalgesia/allodynia after ligation injury. The tendon ligation model developed in the present study attempts to generate a pathological condition with prolonged orofacial nociception of myogenic origin, relevant to myositis of the masseter muscle or myopathies, in general.

Compared to existing preclinical models of myogenic orofacial pain, the unique feature of the present model is its constant and long lasting pain hypersensitivity, thus making it suitable for studying chronicity of pain in TMJ disorders. In addition to inflammation, ligation of the tendon produces mechanical injury and leads to disruption and disarray of collagen fibers, and hypovascularization. The tendinous attachment of the tendon may be further damaged due to contraction, which is unavoidable during mastication. The tendon receives innervation from cutaneous, muscular and peritendinous nerve trunks, although most nerve fibers do not directly reach the main body of the tendon except that Golgi organs located at the junction of the tendon and the muscle receive innervation from large myelinated nerve fibers [33,36,37]. After ligation, networks of nerve fibers and nerve endings on the surface of the tendon may be damaged, which would likely involve unmyelinated nociceptors and autonomic nerve terminals [36].

Ligation of the TASM may lead to biochemical changes in and around the tendon. Cumulating evidence shows that chronic tendinopathy is associated with altered expression of local enzymes, cytokines, and neurotransmitters and receptors [35]. Multiple isoforms of matrix metalloproteinases (MMP) and tissue inhibitors of metalloproteinase are either up- or down regulated [35]. These enzymes contribute to matrix remodeling of injured tendons. However, they may also play a role in generating pain in the tendon. For example, MMP9 that shows increased expression in ruptured tendon has been associated with microglial activation and pain after nerve injury in animal models [38]. Expression of inflammatory mediators cyclooxygenase 2 [39] and interleukin-1 [40] are increased in painful tendon, even without infiltration of inflammatory cells. In tendinopathy patients, chemical mediators of nociception, glutamate and substance P, exhibit increased expression in tendon, along with peripheral NMDA receptors [41-43]. Similarly, following transection injury of the ligament, peptidergic innervation such as substance P and calcitonin gene-related peptide-positive fibers were increased in epiligament tissues in rabbits [44]. These chemical mediators are capable of sensitizing peripheral nociceptors and initiate and maintain hyperalgesia [6,12].

Thus, the factors leading to constant pain after TASM ligation involve cellular changes at the site of injury. They include inflammatory responses, mechanical breakdown of collagen fibers and damage of peripheral nociceptors associated with sensory innervation of the TASM, and changes in local tissue matrix enzymes and neurotransmitters/receptors. The TASM ligation model provides a convenient method to study peripheral neural and immune mechanisms of myogenic pain in TMJ disorders.

Hyperexcitability of central pain processing circuitry may also be involved in long lasting pain after TASM injury. We have chosen a few markers to assess increased excitability of the central trigeminal pain pathways after TASM ligation. Fos protein expression, which is generally absent under normal conditions, has been widely used as a marker of nociceptive neuronal activation [45]. Persistent Fos protein expression has been demonstrated after TMJ inflammation in rats [46]. We identified neuronal activation in the Vc-C1 dorsal horn, the first relay of trigeminal nociceptive input, after TASM injury. The Fos-LI is mainly distributed in the medial-intermediate portion of the superficial laminae, corresponding to V3 input with a V2 component. The masseter muscle is innervated by the masseter nerve from V3 (mandibular nerve). Since the TASM is inserted into the maxilla and tissues in the V2 territory are also injured during ligation of the TASM, V2 (maxillary)-related neuronal activation is observed. Thus, the TASM ligation-induced neuronal activation is somatotopically relevant. Selective induction of Fos-LI in Vc-C1 neurons was consistent with initiation of behavioral hyperalgesia after tendon and tissue injury and suggests nociceptive activation of trigeminal pain pathways. Significant Fos expression was seen at 3 d after ligation. Fos-LI remained increased at 2 weeks, although the level of expression was less than the 3 d time point. This pattern of Fos protein induction is similar to that seen in the TMJ inflammation model and may reflect reduced nociceptive input as tissue repair is progressing. It is notable that at 8 weeks after ligation, there was no significant increase in Fos expression while intense hyperalgesia and allodynia were still present. Interestingly, at 8 w after ligation, no inflammatory cells were observed in the ligated tendon, indicating that tissue inflammation had been resolved. These results suggest that the central hyperexcitability state has been previously established and can be maintained by attenuated primary afferent nociceptive input; or that other down stream targets are involved in the maintenance of the hyperexcitability.

Our results showed enhanced NMDA receptor phosphorylation in trigeminal pain processing circuitry after intraoral injury. The parallel upregulation of P-NR1 and NR1 proteins suggests that *de novo *synthesized NR1 proteins contribute to increased NR1 phosphorylation. Different from the time course of Fos protein expression after TASM ligation, the NMDA receptor NR1 subunit showed constant upregulation in protein translation and phosphorylation throughout the observation period. Astroglial and microglial markers also exhibited increased expression at all time tested. These changes parallel the long-lasting hyperalgesia seen after the tendon ligation and may also be maintained in the face of attenuated primary nociceptive input or by other mechanisms. Information from the sham control animals further confirmed that the levels of NMDA receptor phosphorylation and expression of glial markers returned to the control level at 2 weeks after the sham procedure when hyperalgesia due to tissue injury in sham animals had resolved. These results suggest that the TASM ligation model shares some key neurochemical mechanisms with other models of myogenic and neuropathic orofacial pain in which neuron-glia-cytokine interactions play an important role in central mechanisms of persistent orofacial hyperalgesia [11,21,47-49]. These neurochemical findings indicate that masseter tendon ligation induced long-lasting changes in the trigeminal pain processing circuitry. The parallel or coordinated neuronal and glial responses correlated well with long lasting mechanical hyperalgesia/allodynia after masseter tendon injury and temporary hyperalgesia after intraoral tissue injury due to the sham operation.

We did not observe hyperalgesia contralateral to the ligation, although we did notice a mild hypersensitivity in the ipsilateral TMJ region after TASM ligation (Unpublished observation). This seems consistent with clinical reports that 90% of patients with tenomyositis had a unilateral disorder [31]. The CFA-induced masseter inflammation model, however, produces hyperalgesia remote from the site of inflammation [9,50]. After injecting a relatively large dose of CFA into the masseter muscle, hyperalgesia spreads into the hindpaws of the rat or the robust trigeminal hypersensitivity leads to distant motor activity [50]. We speculate, that compared to tendon ligation, CFA produces an intense inflammatory response that directly activates muscle nociceptors and provides a stronger nociceptive primary afferent input to the CNS. Activation of central pain facilitatory pathways also leads to secondary hyperalgesia [9,19]. Assuming a further diminished role of primary afferent input during the later phase of tendon injury, it would be interesting to determine whether persistence of hyperalgesia seen in the present model relies on a transition from peripheral to central mechanisms. The maintained increase in the expression of glial markers and postsynaptic NMDA receptor phosphorylation that parallels the time course of hyperalgesia lends support to this hypothesis.

## Conclusions

We have developed an orofacial tendon injury model with long lasting pain hypersensitivity of myogenic origin. The TASM is easily approachable and the model is easy to produce. Mechanical hyperalgesia and allodynia of the tendon can be assessed through probing facial skin and are sensitive to clinically used analgesics. The model will be particularly useful in studying the chronicity of pain associated with TMJ disorders. It is worth noting that rats with the tendon injury showed a delayed reduction of body growth, suggesting an effect of the lasting intense hyperalgesia on body health and different mechanisms underlying responses at the early and late phases of tendon injury. Further, the model can also be adapted to other regions of the body for studying pathology of painful tendinopathy seen in sports injury, muscle overuse, and rheumatoid arthritis [51].

## List of Abbreviations

CFA: complete Freund's adjuvant; CNS: central nervous system; ECL: Enhanced Chemiluminescence; EF_50_: the effective force that produces 50% response frequency; GFAP: glial fibrillary acidic protein; LI: like immunoreactivity; MMP: matrix metalloproteinases; NMDA: N-methyl-D-aspartate; PBS: phospho-buffered saline; P-NR1: phosphorylation at serine 896 of the NR1 subunit of the NMDA receptor; S-R curve: stimulus-response frequency curve; Vc: the subnucleus caudalis of the spinal trigeminal sensory complex; TASM: tendon of the anterior superficial part of the rat masseter muscle; TBS: tris-buffered saline; TL: tendon ligation; TMJ: temporomandibular joint.

## Competing interests

The authors declare that they have no competing interests.

## Authors' contributions

WG is involved in the experimental design, performed surgery and Western blot studies. HW is involved in the design and histology and immunohistochemistry experiments. SPZ is involved in behavioral testing and pharmacology experiments. FW contributed to the design of the model and surgical approach. FW, RD and KR conceived of the study, and participated in its design and coordination and helped to draft the manuscript. All authors read and approved the final manuscript.
